# One-Session Versus Three-Sessions of Virtual Reality Exposure Therapy for Public Speaking Anxiety: Protocol of a Randomized Controlled Trial

**DOI:** 10.2196/89612

**Published:** 2026-08-25

**Authors:** Goda Gegieckaitė, Olga Zamalijeva, Karolina Petraškaitė, Egle Nagevice, Philip Lindner, Per Carlbring, Tom Van Daele, Gerhard Andersson, Jonas Eimontas

**Affiliations:** 1 Department of Clinical Psychology, Institute of Psychology, Faculty of Philosophy, Vilnius University, Vilnius, Lithuania; 2 Laboratory of Applied Psychology, Institute of Psychology, Faculty of Philosophy, Vilnius University, Vilnius, Lithuania; 3 Centre for Psychiatry Research, Department of Clinical Neuroscience, Karolinska Institutet, Stockholm, Sweden; 4 Stockholm Health Care Services, Stockholm, Sweden; 5 Department of Psychology, Stockholm University, Psykologiska institutionen Albanovägen 12, Stockholm, 106 91, Sweden, 46 8163920; 6 School of Psychology, Korea University, Seoul, Republic of Korea; 7 Psychology and Technology, Centre of Expertise Care and Well-being, Thomas More University of Applied Sciences, Antwerp, Belgium; 8 Centre for Technological Innovation, Mental Health and Education, Queen’s University Belfast, Belfast, United Kingdom; 9 Department of Behavioural Sciences and Learning, Linköping University, Linköping, Sweden; 10 Department of Biomedical and Clinical Sciences, Linköping University, Linköping, Sweden; 11 Department of Clinical Neuroscience, Karolinska Institutet, Stockholm, Sweden

**Keywords:** public speaking anxiety, virtual reality, exposure therapy, internet-based intervention, randomized controlled trial, anxiety disorders

## Abstract

**Background:**

A 1-session virtual reality (VR) exposure for public speaking anxiety has been found to be effective in reducing public speaking anxiety. Previous meta-analyses comparing 1-session to several sessions of in vivo exposure for specific phobias did not find a difference in effectiveness. However, due to the specificity of VR exposure and public speaking anxiety, it is unclear how dividing the same amount of exposure tasks into 3 shorter sessions instead of one long session would affect the effectiveness of VR exposure for public speaking.

**Objective:**

The aim of this study is to compare the efficacy of a VR exposure-based intervention for public speaking anxiety and the level of VR presence between a 1-session treatment and an equal exposure time divided into 3 sessions, followed by 4 weeks of online intervention in both groups.

**Methods:**

This randomized controlled trial will aim to recruit 86 higher education students experiencing high levels of public speaking anxiety, who will be randomized into one of two conditions: either 1 or 3 sessions of VR exposure therapy, with the same total number of exposure exercises. Both conditions will be followed by the same 4-week online program to encourage in vivo exposure tasks for both conditions. The study will be an exploratory superiority trial testing whether one of the conditions is superior to the other. The primary outcome will be the Public Speaking Anxiety Scale. Assessments will take place pretreatment, at the beginning of the face-to-face session, during the face-to-face session, 1 week after the last VR exposure session, at the end of 4 weeks of the online program, and at two follow-ups at 3 and 12 months after finishing the online program, or equivalent time if the online program was discontinued. Repeated measures within-between ANOVA, the reliable change index (RCI), and effect sizes will be used as statistical analyses, and intention-to-treat will be the primary analysis approach; additional per-protocol analysis will be reported.

**Results:**

The study received funding from the Research Council of Lithuania (LMTLT) in April 2023. Recruitment for the study officially started in January 2024 and was still ongoing during the submission of this paper in December 2025. As of November 2025, a total of 101 participants have completed the initial screening. The final results of the study are expected to be prepared and submitted for publication in March 2026.

**Conclusions:**

This is the protocol of a study that will explore an understudied and clinically important question that could generate preliminary evidence comparing 1-session versus 3-session VR exposure for public speaking and inform further research. However, given the small and specific sample, results would have to be confirmed in larger and more diverse samples.

## Introduction

### Anxiety Disorders and Public Speaking Anxiety

Anxiety disorders are one of the most common mental health problems, with social anxiety disorder (SAD) being found to affect 4%‐12.5% of the general population in their lifetime [1,2]. Social anxiety is the intense fear or anxiety of appearing in front of others (performance), of being observed or judged, or of experiencing shame or public humiliation, and the intensity of the fear causes the individual to avoid threatening social situations and results in disturbances of social, occupational, or other areas [3,4]. Even though many people may experience varying levels of discomfort while speaking in front of others, in severe instances public speaking anxiety may fall under the umbrella of social anxiety disorders in the *DSM-5* (*Diagnostic and Statistical Manual of Mental Disorders* [Fifth Edition]) [3], but it differs from the other SAD subtypes qualitatively and quantitatively, and is therefore distinguished as an independent subtype of SAD [5]. Public-speaking anxiety symptoms can be experienced without SAD, as several previous studies found a much higher lifetime prevalence of significant public speaking fear in community samples (21.2%‐24.8%) compared to the lifetime prevalence of SAD in the same samples (6.6%‐12.1%) [6,7]. However, SAD most often includes a fear of public speaking, with 70.3%‐88.7% of those with SAD also experiencing a significant level of public speaking anxiety [6,7].

Public speaking anxiety is characterized by physiological arousal, negative cognitions, and avoidance or safety behaviors in response to public speaking situations [8]. In cognitive models, public speaking anxiety is caused by negative self-imagery and is associated with overall negative self-perception in social contexts [9]. Individuals with a preexisting negative self-image experience more anxiety in public speaking situations, have more negative thoughts about themselves, and are more critical of their performance [10]. Furthermore, individuals with SAD might have self-imposed high social standards when confronted with an intimidating social situation. They are likely to focus their attention on the anxiety they experience, overestimate the negative consequences of the situation, feel that they have no control over their emotional response, and perceive their social skills as insufficient to cope with the social situation. Public speaking anxiety is furthermore exacerbated by the fear of physiological symptoms [11]. This leads to maladaptive coping—avoidance of engaging in distressing social situations, safety behaviors, and rumination after the event [11]. Avoidance behavior can occur in various contexts and can interfere with adaptation, such as achieving goals in jobs requiring public speaking. For 10% of people, public speaking anxiety causes disruptions in psychosocial functioning or significant distress [12].

### Exposure Therapy for Anxiety Disorders and Public Speaking Anxiety

Exposure therapy has a long tradition of being used for the treatment of anxiety disorders and various phobias [13,14] and has been found to be an effective method for SAD [15] and public speaking anxiety [16] and is often an important part of interventions for public speaking anxiety [17]. Exposure therapy has a variety of forms differing in the structuring of confrontation with the feared situation in the build-up to the intensity of the stimulus, the length of the exposure, and the times in between exposures [18]. Exposure therapy for public speaking anxiety usually involves tasks of giving speeches in front of other people [16]. By continued and repeated exposure to these tasks, a person’s fear response to public speaking should be reduced by the end of treatment. Theories explaining exposure suggest that present anxiety or phobias are a result of an activated fear system, consisting of the feared stimulus, response to it, and meaning of it, which is created by earlier experiences [19,20]. Two main theories slightly diverge in suggesting whether, during exposure, new information showing a lack of threat modifies and extinguishes the existing fear system (the emotional processing model) [19,20] or whether a new safety path, competing with the existing one, is established (an inhibitory learning approach theory) [13,21]. The emotional processing model and an inhibitory learning approach theory are associated with slightly different implications for what processes are the most important during exposure, and sometimes can be traced to different existing traditions and strategies used to maximize exposure therapy effectiveness. Mainly, authors of the inhibitory learning approach, namely, Craske et al [21], criticize the emotional processing model’s emphasis on the need for habituation for the new associations to be created, as previous studies do not confirm that habituation during a session is a good indicator of later fear reduction. Instead, Craske et al [13,21] emphasize the violation of aversive outcome expectations, learning to tolerate anxiety, and the strength of the new association representing safety as more important processes during exposure for more robust long-term exposure therapy effectiveness.

### Virtual Reality Exposure for Public Speaking Anxiety

While exposure therapy is an effective method for anxiety disorders and the process of the method is quite straightforward, the practical execution of exposure in a traditional therapy setting might usually be quite challenging as in vivo exposure is logistically complex [22]. In a traditional therapy session setting, therapists usually will have limited access to patients’ feared stimuli to conduct in-session exposure, even for more common phobias of spiders, dogs, flying, small spaces, etc. This also applies to exposure to public speaking tasks, as setting up an audience specifically for the exposure therapy is ethically and logistically complicated [16]. However, virtual reality (VR) in exposure therapy may help to overcome such limitations. The use of VR is becoming more prevalent in research, as well as in clinical practice [16]. Although less common in the latter setting, the potential of VR seems to be increasing as well, as equipment is becoming more affordable and attitudes of cognitive behavioral therapy clinicians toward VR exposure seem to become more positive [23]. VR addresses ethical and logistical challenges by enabling therapists and patients to work on exposure tasks to a variety of stimuli within the safe and controlled confines of the therapist’s office during regular therapy sessions [24,25]. While there is some discussion about whether exposure in a VR environment can be as effective as in vivo exposure, several meta-analyses showed that VR exposure can be as effective as in vivo exposure for social anxiety disorders [26,27]. A recent meta-analysis [24] found that VR for public speaking anxiety is effective, and another meta-analysis [16] found that while in vivo exposure was slightly more effective, VR exposure was comparably effective to in vivo exposure.

### The Problem of Concentrated vs Spaced VR Exposure Effectiveness

One of the important questions in exposure literature is how to structure exposure to maximize its effectiveness while also optimizing the time and effort demanded from a therapist and a patient. Öst [28] developed a 1-session treatment, an extended 3-hour session with prolonged exposure, primarily aimed at specific phobias. The essential question is whether exposure done in 1 session is as effective as repeated exposure over a longer period of time. Historically, attempts to test the optimal number of exposure sessions and time between sessions for best effects on fear reduction showed mixed results [21]. Different theories would support different strategies for maximizing exposure effectiveness. Traditional habituation and emotional processing theory would emphasize the need for habituation during exposure, which would suggest that longer exposure until a person is no longer feeling anxiety about the stimulus is needed. Öst [29] also describes the development of 1-session treatment based on the idea that a long enough exposure session could achieve full habituation and thus be effective. However, the authors of inhibitory learning theory emphasize that habituation is not necessary and that a more important factor when considering the length and frequency of exposure is maximized variability and unexpectedness of context during exposure to create stronger and more robust new safety associations [13,21]. This would suggest that more frequent exposure in different contexts would be preferable over a prolonged exposure session. Some studies have shown that more than one session showed a better reduction in later fear renewal compared to a 1-session alternative [30,31]. However, a meta-analysis by Odgers et al [32] has found that for specific phobias, a 1-session of in vivo exposure was as effective as treatments with several treatment sessions. As the authors have noted, their meta-analysis was dominated by phobias of animals [32], and as in vivo 1-session treatment has been primarily developed for specific phobias, 1-session exposure treatments might be more suitable for animal phobias than other types of anxiety disorders.

VR exposure introduces an additional dimension to the debate between prolonged and spaced exposure approaches. In VR research, the concept of presence is understood as the extent to which a person connects and engages with the virtual environment [33] or the interpretation of the virtual environment as if it were real [34]. It has been suggested that a sense of presence in a VR environment is necessary for exposure success, as virtual stimuli need to be perceived as real enough to activate the fear system [35]. It might be that presence in a VR environment during exposure might diminish if fear-evoking tasks are performed repeatedly in a short period of time in a single long session. For example, an observed reduction in anxiety might be due to a reduction in presence, but not due to habituation or new safety-representing associations. Factors influencing presence in a VR environment are not widely studied, and the effect of duration in a VR environment on the sense of presence is not clear [36-38]. Research also shows a practical problem of longer VR sessions being associated with increased motion sickness risk [36,39]. This has been found to be associated with increased reaction times and increased heart rate [40], which might negatively impact exposure task effectiveness.

The most commonly mentioned advantage of 1-session treatment is that it offers a clear benefit of saving cost, time, and resources [32]. And of course, for research purposes, the 1-session treatment intervention strategy greatly reduces the issue of participant retention. However, in a traditional therapy setting, a 3-hour session might not be as convenient as suggested, as it is not a traditional time frame for a therapy session; it might be difficult to fit into a regular therapist’s schedule and might require too much time and energy at one time for a therapist and a client. It is possible that the same amount of exposure tasks of one session divided into several sessions might be a more logistically comfortable solution in some settings like traditional therapy or outpatient clinics; however, from previous evidence, it is not clear enough how it would affect the VR exposure for public speaking efficacy.

Due to conflicting theoretical arguments for each condition’s superiority, we hypothesize that the 1-session and 3-session VR exposure for public speaking anxiety will differ in their efficacy without specifying which one will have the superiority. The aim of this study was to compare the efficacy of VR exposure-based intervention for public speaking anxiety and levels of VR presence between two conditions—one-session exposure and the same exposure time divided into 3 sessions, followed by 4 weeks of online intervention for both conditions.

## Methods

### Study Design

The study is a superiority, 2-armed, parallel-group randomized controlled trial. The condition manipulated in the experiment is session spacing—equal VR exposure tasks are either performed in 1 or 3 separate sessions. This protocol follows the SPIRIT (Standard Protocol Items: Recommendations for Interventional Trials) guidelines (Checklist 1).

**Figure 1.**
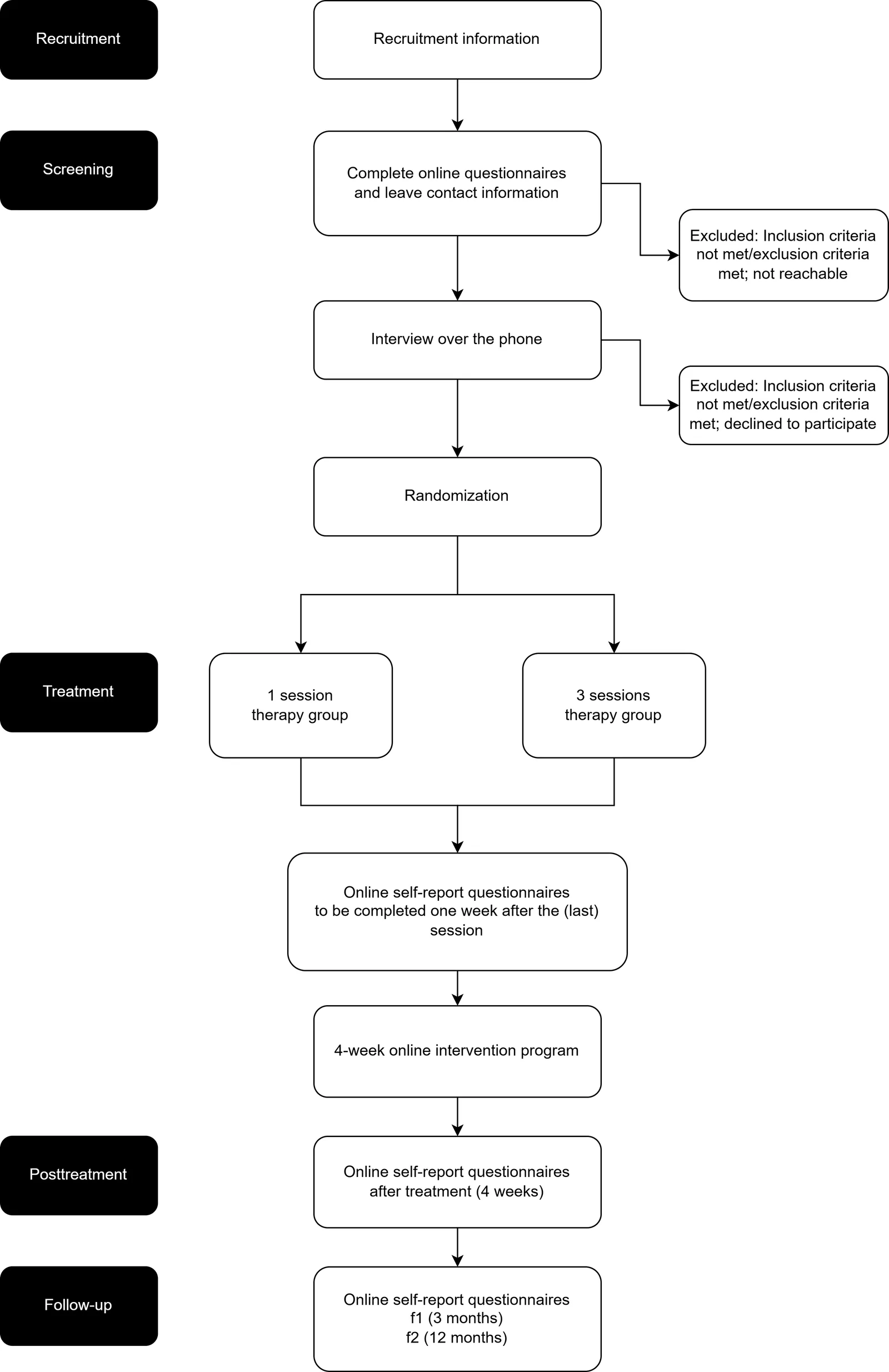
CONSORT (Consolidated Standards of Reporting Trials) diagram for the randomized controlled trial comparing 1-session and 3-session of virtual reality (VR) exposure therapy for public speaking anxiety among young adults.

The study will be conducted in Lithuania. Initial screening for the study and main outcome questionnaires will be completed online. Intake interviews will be done over the phone. The VR exposure part of the intervention will be performed face-to-face on Vilnius University premises. Face-to-face assessments will also be carried out during the in-person session before the start and after the exposure. After the VR exposure sessions, participants will partake in a 4-week online intervention. The participant flowchart is shown in Figure 1.

### Participants and Eligibility Criteria

Participants will be eligible for the program if they meet the following inclusion criteria: (1) young adults aged between 18 and 30 years; (2) students pursuing higher education; (3) a significant level of public speaking anxiety (Public Speaking Anxiety Scale [PSAS] score of 60 or above); (4) access to a computer and internet access for the duration of the study; (5) can understand, write, and speak Lithuanian; and (6) able to participate in 1 or 3 in-person intervention sessions and are able to devote the time to participate in a 4-week online program following the intervention sessions. Participants will be excluded from the study if (1) they have a history of seizures or epilepsy; (2) they have other significant medical conditions that would prevent them from participating in the program; (3) they have high levels of depression (Patient Health Questionnaire-9 [PHQ-9] rating of 15 and above and mentions of suicidal ideation) or other significant psychiatric conditions that would interfere with participation in the program; (4) they have a tendency to have extreme motion sickness reactions or a history of negative physical reactions to VR experiences or difficulty with or lack of stereoscopic vision; (5) they are currently involved in other psychological interventions such as psychological counseling or psychotherapy; (6) they use psychoactive drugs, unless stable for 3 months; or (7) they are currently participating in other programs aimed at reducing public speaking anxiety.

### Sample Size

Sample size was calculated for superiority analysis to test whether one condition shows bigger effects at least at the medium effect level. Power analysis was performed using the G*Power program for repeated measures ANOVA between factors’ effects, which showed that given a significance level of .05 to achieve 80% power to detect a medium between-groups effect (Cohen *f*=0.25) with 2 groups × 3 time measurements (pretreatment, 1 week post last exposure session, and at the end of the online intervention period or equivalent time), with an estimated correlation between repeated factors of 0.5, a total sample of 86 is needed.

### Recruitment

The recruitment process will focus on Lithuanian-speaking students who are able to participate in in-person sessions on Vilnius University premises. The information about the study will be shared using different sources and different media outlets. First of all, the information will be sent out through emails, using student email accounts. Moreover, social media posts will be published on official social media accounts administered by the administration of higher education institutions, as well as student organizations of said institutions. Student organizations will also be asked to share information about the study in informal student social media groups. Paper posters will be put up in the common areas of higher education institutions. Finally, the representatives of the research team will seek to participate in various informational events at the higher education institutions and present in person the opportunity to participate in said study.

### Assignment of Interventions: Allocation

A random number generator (random.org) will be used to allocate participants randomly and equally to the study groups. A blocked randomization will be used as participants’ inclusion in the study will continue. Team members not involved in the participants’ screening and interviewing will generate a number and allocate participants to one of the two groups. A team member randomizing and allocating participants to one of the groups will have no information about the participant other than a generated user code.

### Implementation

Participants will be randomized and allocated to one of the two groups by team members who will not be involved in participants’ assessments and phone interviews. Randomized lists for allocation to two groups will be generated in advance with 3 batches of 20 blocked allocations and 3 batches of 10 blocked allocations at a time through a random number generator (random.org) by a team member not conducting preintervention assessments and phone interviews. During phone interviews, participants who indicated high levels of depression and suicidal ideation during screening will be informed about exclusion and provided with detailed information about available professional mental health services. After a participant is admitted to the study following a phone interview, a team member responsible for conducting phone interviews will provide a system-generated user code to the team member responsible for participants’ allocations, who will not have any other information about the participant. This allocator will then add the new participant to the existing randomized list of allocations to two groups in the sequential order of admittance to the study, which will assign them to one of the two groups.

### Assignment of Interventions: Blinding

Because of the nature of the intervention, neither participants nor therapists can be blinded to the intervention conditions. However, participants will not be explicitly told that the effect of the number of exposure sessions is tested. In the informed consent, participants will be informed that they will be asked to participate in up to 3 sessions of VR exposure, and after allocation to one of the groups, the participants will only be asked to participate in either 1 or 3 exposure sessions. Screening and posttreatment outcomes assessment will be done online, which will eliminate assessor bias. However, participants will be asked to again complete the primary outcome questionnaire in person right before the start of the exposure session and will complete some assessments during the session and right after.

### Interventions

Some studies have found that 1 session of VR exposure plus 4 weeks of an online program for public speaking anxiety was effective in reducing public speaking anxiety [25,41]. A previous meta-analysis comparing one session to several sessions of in vivo exposure for specific phobias did not find a difference in effectiveness between the two [32]. However, because of the specificity of VR exposure and public speaking anxiety, it is unclear how dividing the same amount of exposure tasks into three 1-hour-long sessions, including 3 tasks each (which is a more typical duration for a therapy session), instead of one 3-hour-long session including a total of 9 tasks, would affect the effectiveness of VR exposure for public speaking. After the exposure sessions, a 4-week online program to encourage the transition to in vivo exposure for public speaking will be part of both conditions.

The VR exposure intervention for public speaking anxiety chosen for this study is based on established tasks and negative belief testing used in traditional cognitive behavioral therapy and exposure-based interventions for public speaking anxiety. The effectiveness of this type of intervention has been confirmed in previous studies [42,43]. The intervention consists of two parts: a face-to-face VR exposure session, which has been adapted and modified from a previous study by Lindner et al [43], and a 4-week online intervention developed by Lindner et al [43]. Participants will be randomized into two intervention groups, either consisting of 1 or 3 VR exposure sessions, cumulatively consisting of the same number of same-length exposure tasks. Both groups will participate in a 4-week online transition intervention after VR sessions.

### VR Exposure Sessions

At the beginning of the VR exposure session, all participants will be presented with psychoeducation on public speaking anxiety and the principles of exposure tasks. Over the course of the session, participants will perform 9 exposure tasks that will last from 1 minute to 3 minutes each. Two of these 9 speaking tasks will have 1 and 2 additional minutes for preparation before the speech. During exposure tasks, participants will use a VR headset to virtually stand before varying-sized audiences and distances from the crowd (specific audiences will be consistently ascribed to specific tasks). Tasks will vary in difficulty and will include participants having to count backward, come up with as many words as possible starting with the same letter, talking about themselves, their interests, achievements, their fear of public speaking, and preparing and giving a speech on one of the several controversial topics like the admission of migrants, euthanasia, abortion, etc. Concerning the controversial topics, participants will be allowed to pick one out of a list of topics to reduce unnecessary discomfort or will be able to refuse to complete the task, based on the voluntary participation condition described in informed consent. Similar to Lindner et al [25,43] in-session procedure, before each task, participants will be asked to report their level of distress, identify their negative beliefs/expectations about each public speaking task, and evaluate the chances of their negative expectations to come true. Their performance of the task will be recorded. After each task, participants will rate the current level of distress, the highest level of distress during the task, the perceived quality of their performance, and the level of presence they felt in the VR environment. Subsequently, participants will be invited to listen to the recording of their speech and instructed to imagine that they are listening to this speech from a third-person perspective while in the same audience. Afterward, they will again be prompted to evaluate the speech quality and the degree to which their negative beliefs or expectations were confirmed. At the end of the session, participants will be asked to provide feedback on the convenience of the 3-hour 1-session format, and they will be given information about the importance of the transition to in vivo exposure in their daily lives and encouraged to use online intervention to help them with the in vivo exposure transition.

In the group of the 3 VR exposure sessions, the same procedure will be applied, except that the same exposure tasks in the same order will be divided into the 3 sessions (first session: tasks 1‐3; second session: tasks 4‐6; third session: tasks 7‐9). All 3 sessions will be held over a period of 2 weeks. Psychoeducation will be provided only in the first session, and information about the importance of transition to in vivo exposure as well as an assessment of the convenience of the 3-session format will be done only in the last session. Exposure sessions will be conducted by trained clinical and health psychologists and psychology master’s students supervised by experienced psychologists.

### Online Intervention

A self-guided online intervention that has been developed and studied in previous VR exposure studies by Lindner et al [25,43] will be used. The intervention was adapted and translated into Lithuanian in collaboration with the intervention developers. One week after the last VR session, participants will be invited to complete a postexposure assessment, after which they will be able to start the online transition intervention.

The online intervention consists of 4 modules, each available consecutively over the course of 4 weeks. The modules reiterate important psychoeducational information and focus on encouraging the participants to plan and carry out public speaking in vivo exposure tasks. They are asked to complete 4 public speaking tasks per week, for example, to attend an event or presentation and ask questions, to do a public presentation, or to pretend to speak on the phone on a bus or train [43]. Each of the 4 modules is designed to work on important aspects of overcoming public speaking anxiety: the first module introduces the transition from VR exposure tasks to public speaking in everyday life, the second module focuses on safety behaviors, the third module concentrates on catastrophic beliefs, and the fourth module covers the planning for the future and relapse prevention [43]. If participants have any questions about the tasks of the online program during these 4 weeks, they will be able to contact the program psychologist through the online program’s secure messaging system. A so-called therapist support-on-demand approach was chosen for this study.

### Criteria for Discontinuing or Modifying Allocated Interventions

It is in the nature of the exposure tasks that participants are expected to experience some level of distress, and it is important not to discontinue exposure because of experienced anxiety. However, if participants experience significant distress and, after careful consideration and discussion, participants still do not feel comfortable continuing, exposure tasks will be suspended. In this case, emotional support will be provided by the therapist conducting the session. Participants might be referred for further psychological help to psychological services. Intervention may be discontinued if participants express significant discomfort and wish to discontinue for other reasons, for example, because of motion sickness. All cases of discontinuation and the reasons would be recorded and reported. During the online intervention, participants will be instructed to notify the research staff in case of a significantly deteriorating psychological condition. In that case, a trained psychologist from the team will conduct a clinical assessment via telephone interview. Based on the evaluation, the participant may be referred to alternative psychological services.

### Strategies to Improve Adherence to Interventions

To improve adherence to the intervention, participants will be contacted by phone, first discussing whether participants will be able to participate in the exposure sessions, as well as getting confirmation that they will be able to participate in the 4-week online intervention. If patients cancel or do not show up to their scheduled exposure sessions, they will be contacted via phone to discuss possible barriers and, if possible, schedule a new suitable appointment for a session. However, if they express the desire not to participate in the study, they will be able to do so freely. Finally, participants who do not log into the online intervention will also be contacted to provide encouragement and technical support for them to be able to participate.

### Outcome Measures

#### Primary Outcome Measures

To assess public speaking anxiety and to track the changes after the intervention, the PSAS [44,45] will be used. Assessment with the PSAS will take place at preintervention (during participant selection), at the beginning of the first exposure session, postintervention at week 1, after finishing the online program at week 5, and two follow-ups at 3 months and 12 months after finishing the online program. The PSAS score during online eligibility screening will be the baseline measure; change in score from baseline will be assessed. The scale consists of 17 self-report statements (eg, “giving a speech is terrifying”) measured on a Likert-type scale ranging from 1 “not at all” to 5 “extremely.” This scale measures 3 components of public speaking anxiety: cognitive (8 items), behavioral (4 items), and physiological (5 items). The overall score on the scale ranges from 17 to 85. A higher score indicates higher levels of experienced public speaking anxiety. Initial assessment of the factor structure and reliability of the Lithuanian version of PSAS was conducted with 227 participants aged 18‐25 years. The scale showed good internal consistency (α=.93), while factor structure analysis supported the use of the total score (root-mean-square error of approximation [RMSEA]=0.07), suggesting it is suitable for use in the young adult sample [45].

#### Secondary Outcome Measures

The severity of participants’ social anxiety will be assessed with a self-report version of the Liebowitz Social Anxiety Scale (LSAS-SR) [46,47]. Assessment with the LSAS-SR will take place at preintervention, postintervention at week 1, after finishing the online program at week 5, and during follow-ups at 3 and 12 months. A change in score from baseline will be assessed. The LSAS-SR is a 24-item scale that measures fear or anxiety and avoidance of different social situations over the past week. It consists of 11 items addressing social interaction situations (eg, “meeting strangers”) and 13 items addressing public performance situations (eg, “acting, performing, or giving a talk in front of an audience”). Each item is rated twice using 4-point Likert scales, one for the intensity of anxiety ranging from 0 “none” to 3 “severe” and the other one for the frequency of avoidance of the situation ranging from 0 “never” to 3 “usually.” An overall score is calculated by summing the anxiety and avoidance ratings for all 24 items, with results ranging between 0 and 144. LSAS-SR also allows the calculation of anxiety and avoidance subscales, where each can be divided into 2 subscores: social interaction anxiety and performance anxiety within the anxiety subscale, and social interaction avoidance and performance avoidance within the performance subscale. A higher score indicates more severe symptoms. To assess the eligibility of the Lithuanian version of LSAS-SR, a pilot study was conducted with 168 participants aged 18‐25 (mean age 21.01, SD 2.67) years. The scale showed good internal consistency (α=.92 to .95) and sufficient construct validity (RMSEA=0.06) and is suitable for use in the young adult sample.

Individuals’ level of apprehension regarding the possibility of receiving negative evaluations will be assessed using a self-report questionnaire, the Brief Fear of Negative Evaluation Scale (BFNE) [48]. Participants will be instructed to complete the questionnaire preintervention, postintervention at week 1, after finishing the online program at week 5, and during follow-ups at 3 and 12 months. The BFNE is a 12-item, 5-point Likert-type scale, ranging from 0 “not at all” to 4 “extremely.” The statements in the scale describe the presence or absence of fear or worrying about being negatively regarded by others (eg, “I am frequently afraid of other people noticing my shortcomings”). Individuals who score high on the BFNE exhibit a heightened fear of negative evaluations, a characteristic associated with social anxiety. To circumvent the prospects of being unfavorably judged, individuals with high scores tend to adjust their behavior accordingly [48,49]. The study will use a validated Lithuanian version of the BFNE. According to a previous study [50], the internal consistency of this version is satisfactory (α=.85). Additionally, a previously mentioned pilot study also included the Lithuanian version of the BFNE, revealing good internal consistency (α=.94) and adequate construct validity (RMSEA=0.04).

#### Other Outcome Measures

##### In-Session Assessments

A series of single-item measures will take place before and after the speaking tasks, as well as following the review of the audio recording. Each measure will be rated on a scale from 0 to 100, but using different verbal anchors at the extremes. The subjective level of distress (using the Subjective Units of Distress Scale [SUDS]), rated from “no distress” to “extremely high distress,” will be measured once the speaking task is presented to the participant and twice after the task is completed, asking the participant to indicate the SUDS after the task and the highest level reached during the task. Participants will also be asked to indicate their negative beliefs and expectations related to the task; that is, they will be asked to provide a qualitative description of their fears and concerns related to upcoming task performance, and then they will also be asked to rate the likelihood of these fears and concerns to become true from “definitely not going to happen” to “definitely going to happen,” and after the task was completed and the audio recording was reviewed, participants will once again rate to which extent their expectation has proven to be true from “not true at all” to “completely came true.” Furthermore, participants will be asked to rate the quality of their performance from “completely failed” to “completely succeeded” right after each task and following the review of the audio recording.

The sense of presence will be measured in two different ways. First, after each task, using a scale from 0 “definitely not” to 100 “definitely yes,” participants will be asked to rate whether they actually had the sense of “being there” while performing the speaking task in VR. Second, to assess the overall experience, the sense of presence of participants will be measured with the Igroup Presence Questionnaire (IPQ) [51]. Assessments will be taken after a 1-session VR exposure treatment in the 1-session condition or after each session in the 3-session exposure condition, and participants will be asked to answer questions about their sense of presence during the whole session. This questionnaire consists of 14 items measuring 3 subscales: spatial presence, involvement, and experienced realism. Each item is rated on a 7-point Likert scale, and the score is calculated by summing the ratings for each item. The scale previously showed good psychometric properties (α=.87) [52].

At the end of the 1-session format or before closing the last of the 3-session format, participants will be asked to evaluate the format of the intervention they participated in. Using a 7-point semantic differential type scale, the participants will be asked to rate their attitude and experience regarding 5 characteristics: convenience, duration, fatigue, intensity, and adaptability. The two groups will receive slightly different and specific parallel wording in the instructions. For the 3-session format, “You met the researcher three times and each session took about an hour, how would you rate that format?” while for the 1-session format, “You met the researcher one time and this session took about three hours, how would you rate that format?”

##### Other Measures

The PHQ-9 [53] will only be used for screening purposes during the participant selection stage and will serve as one of the exclusion criteria. PHQ-9 is a short but reliable tool to assess the severity of depressive symptoms. PHQ-9 consists of 9 items that are rated depending on how often the symptoms were bothersome using a 4-point scale, ranging from 0 “not at all” to 3 “nearly every day.” The ratings are summed for the total score, and a score of 15 and above indicates a risk of severe depression [54]. The Lithuanian version of the PHQ-9 has been validated and showed good psychometric properties (α=.86) in a young adult sample [55].

Treatment satisfaction and negative effects will be assessed posttreatment after finishing the online program at week 5. Participants will be asked to answer questions about treatment (both VR exposure sessions and online program) satisfaction and report negative effects as well as rate the level of these effects. Treatment engagement will be measured by the number of modules opened and will be analyzed descriptively. Treatment expectancy will also be measured and used for secondary data analysis.

### Data Collection and Management

#### Plans for Assessment and Collection of Outcomes

Data will be collected at prerandomization, during the sessions, 1 week after the last session before starting the online intervention, after 4 weeks of online intervention, and then at 3- and 12-month follow-ups. Baseline assessment, posttreatment assessment, and follow-up assessments will be completed online. At the beginning of the exposure session, participants will fill in the primary measure again using a paper-and-pencil questionnaire format, and in-session assessments will be filled in the paper session protocol as well. All questionnaires will be based on participants’ self-report. The full timeline of assessments is provided in Table 1.

**Table 1.** Schedule of enrollment, interventions, and assessments for the randomized controlled trial comparing 1-session and 3-session of virtual reality (VR) exposure therapy for public speaking anxiety among young adults.

Assessment/activity	Screening		Postrandomization						Follow-up	
	*t*_−1_ ^a^	*t*_0_ ^b^	*t*_1_ ^c^			*t*_2_ ^d^	*t*_3_-*t*_6_^e^	*t*_7_ ^f^	*f*_1_ ^g^	*f*_2_ ^h^
			Pre-VR exposure	During exposure	Post-VR exposure				3 months	12 months
Informed consent	✓		✓							
Demographic data	✓									
LSAS-SR^i^	✓					✓		✓	✓	✓
PSAS^j^	✓		✓			✓		✓	✓	✓
BFNE^k^	✓					✓		✓	✓	✓
PHQ-9^l^	✓									
IPQ^m^					✓					
In-session assessments: SUDS^n^, catastrophic belief expectancy, quality of performance, single-item presence measure				✓						
Evaluation of intervention format (1-session or 3-session)					✓					
Treatment satisfaction, negative effects								✓		
VR exposure therapy				✓						
4-week online program							✓			
Eligibility criteria	✓	✓								
Treatment expectancy	✓									
Treatment engagement							✓			

a *t*_−1_: online eligibility screening and control data collection (baseline).

b *t*_0_: further screening during telephone interview.

c *t*_1_: face-to-face VR exposure intervention delivery, either via one 3-hour session or via three 1-hour sessions.

d *t*_2_: outcome assessment 1 week after completion of face-to-face VR exposure intervention, either after one 3-hour session or after three 1-hour sessions (first primary endpoint).

e *t*_3_-*t*_6_: 4 weeks of online program with new tasks and materials available weekly.

f *t*_7_: outcome assessment after finishing the 4-week online program or equivalent time if the online program was discontinued (second primary endpoint).

g *f*_1_: follow-up in 3 months after finishing the online program or equivalent time if the online program was discontinued.

h *f*_2_: follow-up in 12 months after finishing the online program or equivalent time if the online program was discontinued.

i LSAS-SR: Liebowitz Social Anxiety Scale – Self-Report.

j PSAS: Public Speaking Anxiety Scale.

k BFNE: Brief Fear of Negative Evaluation Scale.

l PHQ-9: Patient Health Questionnaire-9.

m IPQ: Igroup Presence Questionnaire.

n SUDS: Subjective Units of Distress Scale.

#### Plans to Promote Participant Retention and Complete Follow-Up

Participants will be informed about the follow-up assessments prior to the study. Participants will need to confirm that they are willing and able to participate in face-to-face sessions and online intervention before being included in the study. Simple reminders to fill out the follow-up questionnaires will be sent by email, followed by a phone call. In case participants discontinue the intervention, team members will contact them to first address any concerns that might be interfering with the intervention continuation.

#### Data Management

All of the data will be collected and stored on a secure, encrypted server of the Iterapi platform [56]. For data analysis, data will be kept on password-protected team members’ computers. Personal data like contact information will only be stored throughout the duration of the study and follow-ups.

### Statistical Analysis

The primary outcome will be a change in PSAS scores from baseline to posttreatment assessment (two primary endpoints: *t*_2_ and *t*_7_), as well as during follow-up assessments in both intervention groups. Secondary outcomes will be changes in secondary outcome measures from baseline to posttreatment and follow-ups. Repeated measures within-between ANOVA and effect sizes will be used to assess and compare outcomes within and between groups. The reliable change index (RCI) will be used as a supplementary analysis to analyze individual outcomes following intervention. No interim analyses or formal stopping rules were planned because this intervention is considered low-risk and is not expected to cause any harm to the participants. Regression analysis will be used to test the extent to which the level of anxiety and other psychological states measured by primary and secondary measures during baseline, sense of presence (IPQ at the end of the treatment), age, and gender, and user experience rated by participants predict the efficacy of the intervention. Single-item sense of presence after each task and user experience ratings of the format will be used for further exploratory purposes. In order to handle protocol nonadherence and missing data, it is planned that intention-to-treat will be the primary analysis approach, supplemented by additional per-protocol analysis. As recommended by the CONSORT (Consolidated Standards of Reporting Trials) guidelines, both analyses will be performed and reported for primary outcomes [57], allowing comparison of the effects of both approaches. Multiple imputation techniques will be used to fill in missing data.

### Ethical Considerations

The trial was approved by the Committee on Research Ethics in Psychology at Vilnius University on November 13, 2023, no. 21/(1.13 E) 250000-KT-166 Vilnius. All participants will be informed about the study conditions and will have to sign an informed consent form before participating in the study.

Informed consent will be collected using an online form before starting the initial screening. Additional written informed consent to participate in the intervention will be obtained face-to-face before the beginning of the VR exposure sessions by the researcher conducting the session. Participants will provide their contact information—email and telephone number—which will be stored in a secure server that will be available to access only to the research team after logging in to the platform. Each participant will have a generated ID number that will be used for appointment scheduling and as a stable identifier in the database across measurements. Signed informed consent forms that also include the name of the participant and contact information will be stored in a locked safe that can only be accessed by one of the team members.

The day-to-day operations will be overseen by the principal investigator with the support of the Lithuanian research team. The trial will receive regular and need-based support from Swedish and Belgian researchers who have vast experience in online-based interventions and VR applications for treating various psychological issues. In case of a need for major changes in the trial protocol, these changes will be reviewed and approved by the institution’s Research Ethics Committee before being implemented. No separate data monitoring committee will be appointed in this study. Participants’ experiences and fidelity to the protocol in the course of face-to-face sessions will be discussed weekly and reported by therapists performing the sessions during the supervision meetings. During the online intervention, participants will be instructed to contact the program’s therapists in case of a deteriorating psychological condition. During the period of data collection, the principal investigator will meet weekly with the research team and the therapists to review the progress and discuss any issues.

Participants will be instructed to reach out via the online intervention platform or other means to the research team in case they experience adverse effects or a worsening of psychological state after the face-to-face session or during the online intervention program. All incidents of adverse effects will be reported.

## Results

### Recruitment

The recruitment to the study officially started in January 2024. As of November 2025, a total of 101 participants have completed the initial screening, of whom 37 met the inclusion criteria and were randomized. Recruitment of participants was still ongoing during the submission of this paper in December 2025. The final results of the study are expected to be prepared and submitted for publication in March 2026.

### Protocol Version History

The study’s design has been registered, and protocol version 1 was prepared and submitted to the journal before the start of the participants’ recruitment. The review process has encountered significant delays—16 months for the first decision postreviews and an additional 3 months postrevisions before the manuscript was withdrawn and resubmitted to JMIR. This resulted in the protocol not being published before the end of the study. However, this protocol version 1.3 does not include any changes to planned study procedures and analysis from protocol version 1 submitted before the start of the study, and the only revisions made to the protocol were more detailed information and explanations of the mentioned procedures and plans. No changes to the analysis plans in the protocol were made after the start of data analysis. One change was made in the registration and protocol at the beginning of the study—the inclusion criteria were changed from young adults in bachelor’s studies to young adults pursuing higher education. Explanations of the main changes in different revisions of the protocol are shown in Textbox 1.

Textbox 1. **Protocol version changes.**

**Version 1 (January 25, 2024)**

**Version 1.1 (July 31, 2025):** More detailed information on recruitment strategies, plans to promote participant retention, supervision, protocol amendment communication, an explanation of why no interim analysis or data monitoring committee is planned, and some theoretical introduction revisions.

**Version 1.2 (December 15, 2025):** Protocol version 1.1 is reworked to the format required by the journal. The content of the protocol is not changed from protocol version 1.1.

**Version 1.3 (July 3, 2026):** No major changes to the plan of the study, but some of the details are more clearly stated, for example, discontinuation of the VR sessions if participants state significant discomfort for any reason, not only because of the exposure distress; explicitly stating the study is a superiority trial; clarifying which measure of presence and program engagement is used, and that they will be used for secondary and descriptive analysis. The analysis plan was not changed at this point, except for some more explicit clarifications.

## Discussion

### The Importance of Different Formats

This study will aim to test whether 1-session VR exposure effectiveness for public speaking anxiety differs from the same amount of exposure tasks divided into 3 sessions over the period of 2 weeks, while both programs include 4 weeks of online intervention after the exposure sessions. We consider this study to address an important and practical question regarding the optimal arrangement of VR exposure therapy delivery for public speaking anxiety. A few previous studies found that one VR exposure session [25,43] and 4 sessions of VR exposure therapy [42] were effective in reducing public speaking anxiety. However, to our knowledge, no previous study directly compared the same exposure tasks performed in 1 session and divided into 3 sessions in the context of VR exposure for public speaking anxiety. One session of exposure therapy might be convenient in some contexts but also might be too intensive and long for traditional therapy settings. Extended VR usage in a short period of time might also introduce additional disadvantages in a 1-session exposure. On the other hand, 1-session exposure therapy might be convenient in some contexts, where the number of possible appointments is limited. In addition, the chances of discontinued treatment are likely to be higher in multiple VR exposure sessions compared to one session. This study will compare exposure tasks divided into 3 shorter sessions and a 1-session VR exposure treatment. The study will test an understudied and clinically important question that could generate preliminary evidence of 1-session versus 3-session VR exposure for public speaking comparison and would have the potential to inform future research into optimizing VR exposure for public speaking. However, given a quite limited and specific sample, results would have to be confirmed in larger and more diverse samples.

### Limitations

There are several limitations and risks that we anticipate during the trial. First of all, exposure therapy, by the nature of the method, induces anxiety and discomfort. Participants might be more reluctant to participate in the study and drop out after one exposure session. There will be a need to evaluate the discomfort level of the participant and encourage continuation of the exposure even when they experience discomfort; however, in severe cases of negative reactions, exposure will be discontinued. Another problem we anticipate is participants’ reluctance to continue a transition to in vivo exposure during 4 weeks of online intervention. Low compliance was reported in the equivalent program after VR exposure in the previous study by Lindner et al [43]. To increase compliance, we will ask participants to confirm they will be able to participate in the 4-week online program after the exposure by emphasizing the importance and rationale of online intervention during the in-person session. Another limitation is that although we will measure presence and hypothesized that the length of the sessions might be related to cybersickness for some, for a more parsimonious design of the study, other than excluding participants who reported a history of strong motion sickness, we will not explicitly measure and test motion sickness during VR exposure sessions, which could be one of the confounders of the exposure effectiveness between the different conditions. Due to a very specific and convenient sampling strategy in this research, generalization of the obtained results is strictly limited to young adults pursuing higher education and cannot be directly applied to clinical populations or people from other age groups or social categories. Yet another limitation is that this research focuses on the comparison of different delivery methods with the same amount of VR exposure followed by an online program. Since it does not include a control group that does not have an active intervention, this research is not testing whether this VR exposure therapy is more effective than no treatment, treatment as usual, or a stand-alone online program, but explores comparative efficacy.

Another limitation is that the original protocol version did not detail some of the statistical analysis plans such as handling of sphericity/normality violations, missing data, and multiple imputation model variables, and correction for multiple comparisons. Repeated measures ANOVA might not be the most suitable analysis for the study questions, and a more robust method such as linear mixed effects modeling could be a more appropriate statistical analysis. Moreover, the initially planned multiple primary endpoints have been deemed complicated; thus, *t*_2_ (ie, outcome assessment 1 week after completion of face-to-face VR exposure intervention, either after one 3-hour session or after three 1-hour sessions) is now being considered as the main primary endpoint because the assessment is performed following different conditions, while *t*_7_ remains an additional primary endpoint. We could not add these to the protocol’s statistical analysis plan because this was pointed out by the reviewers after the research team had already seen and worked with the data. These final statistical decisions, including considerations to include more robust analysis methods, will be described in the reports of the study results.

### Conclusions

This protocol details the study that will explore whether a 3-session exposure treatment differs in efficacy from a 1-session treatment for VR exposure–based treatment of public speaking anxiety. This question has relevance in research and practice and could provide preliminary evidence for comparison of different VR exposure for public speaking spacing and scheduling efficacy that could inform future larger confirmatory trials.

## Supplementary material

10.2196/89612 Checklist 1 SPIRIT checklist.

## Abbreviations

BFNE: Brief Fear of Negative Evaluation Scale
CONSORT: Consolidated Standards of Reporting Trials
*DSM-5*: *Diagnostic and Statistical Manual of Mental Disorders* (Fifth Edition)
IPQ: Igroup Presence Questionnaire
LMTLT: Research Council of Lithuania
LSAS-SR: Liebowitz Social Anxiety Scale – Self-Report
PHQ-9: Patient Health Questionnaire-9
PSAS: Public Speaking Anxiety Scale
RCI: reliable change index
RMSEA: root-mean-square error of approximation
SAD: social anxiety disorder
SPIRIT: Standard Protocol Items: Recommendations for Interventional Trials
SUDS: Subjective Units of Distress Scale
VR: virtual reality
