# Supplementary material for: One-Session Versus Three-Sessions of Virtual Reality Exposure Therapy for Public Speaking Anxiety: Protocol of a Randomized Controlled Trial
Source: JMIR Res Protoc. 2026 Aug 25;15:e89612. doi: 10.2196/89612 (PMC13505604; doi:10.2196/89612)
Supplement: Supplementary file 1 [file resprot-v15-e89612-s001.docx]

SPIRIT checklist of items to address in a randomized trial protocol.

| Title {1} | One-session versus three-sessions of virtual reality exposure therapy for public speaking anxiety: study protocol of a randomized controlled trial |
| --- | --- |
| Trial registration {2a and 2b}. | This study has been registered in clinicaltrials.gov. Clinical‑ Trials.gov Identifier:  NCT06214039  <https://clinicaltrials.gov/study/NCT06214039> |
| Protocol version {3} | Protocol version 1. January 1, 2024. |
| Funding {4} | This project has received funding from the Research Council of Lithuania (LMTLT), agreement No S-MIP-23-86. |
| Author details {5a} | Goda Gegieckaitė, PhD  Department of Clinical Psychology, Institute of Psychology, Faculty of Philosophy, Vilnius University, Vilnius, Lithuania, [goda.gegieckaite@fsf.vu.lt](mailto:goda.gegieckaite@fsf.vu.lt)    Olga Zamalijeva, PhD  Laboratory of Applied Psychology, Institute of Psychology, Faculty of Philosophy, Vilnius University, Vilnius, Lithuania, [olga.zamalijeva@fsf.vu.lt](mailto:olga.zamalijeva@fsf.vu.lt)    Karolina Petraškaitė, PhD  Department of Clinical Psychology, Institute of Psychology, Faculty of Philosophy, Vilnius University, Vilnius, Lithuania, [karolina.petraskaite@fsf.vu.lt](mailto:karolina.petraskaite@fsf.vu.lt)    Eglė Nagevičė  Department of Clinical Psychology, Institute of Psychology, Faculty of Philosophy, Vilnius University, Vilnius, Lithuania, [egle.nagevice@gmail.com](mailto:egle.nagevice@gmail.com)    Philip Lindner, PhD  1) Centre for Psychiatry Research, Department of Clinical Neuroscience, Karolinska Institute, Stockholm, Sweden,  2) Stockholm Health Care Services, Region Stockholm, Stockholm, Sweden,  [philip.lindner@ki.se](mailto:philip.lindner@ki.se)    Per Carlbring, PhD  Department of Psychology, Stockholm University, Stockholm, Sweden,  [per.carlbring@psychology.su.se](mailto:per.carlbring@psychology.su.se)    Tom Van Daele, PhD  1) Psychology and Technology, Centre of Expertise Care and Well-being, Thomas More University of Applied Sciences, Antwerp, Belgium,  2) Centre for Technological Innovation, Mental Health and Education, Queen’s University Belfast, United Kingdom,  [tom.vandaele@thomasmore.be](mailto:tom.vandaele@thomasmore.be)    Gerhard Andersson, PhD  1) Department of Behavioural Sciences and Learning, Linköping University, Linköping, Sweden,  2) Department of Biomedical and Clinical Sciences, Linköping University, Linköping, Stockholm, Sweden,  3) Department of Clinical Neuroscience Karolinska Institute, Stockholm, Sweden,  [gerhard.andersson@liu.se](mailto:gerhard.andersson@liu.se)     Jonas Eimontas, PhD  Department of Clinical Psychology, Institute of Psychology, Faculty of Philosophy, Vilnius University, Vilnius, Lithuania, [jonas.eimontas@fsf.vu.lt](mailto:jonas.eimontas@fsf.vu.lt) |
| Name and contact information for the trial sponsor {5b} | Vilnius University  3 Universiteto St., LT-01513 Vilnius  Phone: +370 5 268 7000  E-mail: infor@cr.vu.lt |
| Role of sponsor {5c} | Sponsor and funder have no role in study design, collection, management, analysis, and interpretation of data; writing of the report. Principle investigators have full control of the decision to submit the report for publication. |

*Note*: the numbers in curly brackets in this protocol refer to SPIRIT checklist item numbers. The order of the items has been modified to group similar items (see http://www.equator-network.org/reporting-guidelines/spirit-2013-statement-defining-standard-protocol-items-for-clinical-trials/).
